# Introduction of birth dose of hepatitis B virus vaccine to the immunization program in Ethiopia: an economic evaluation

**DOI:** 10.1186/s12962-020-00219-7

**Published:** 2020-07-22

**Authors:** Solomon Tessema Memirie, Hailemichael Desalegn, Mulugeta Naizgi, Mulat Nigus, Lisanu Taddesse, Yared Tadesse, Fasil Tessema, Meseret Zelalem, Tsinuel Girma

**Affiliations:** 1 Department of Pediatrics and Child Health, College of Health Sciences, Addis Ababa University, Addis Ababa, Ethiopia; 2 Department of Global Public Health and Primary Care, University of Bergen, Bergen, Norway; 3 Department of Internal Medicine, St. Paul’s Hospital Millennium Medical College, Addis Ababa, Ethiopia; 4 Department of Pediatrics and Child Health, College of Health Sciences, Mekelle University, Mekelle, Ethiopia; 5 Federal Ministry of Health of Ethiopia, Addis Ababa, Ethiopia; 6 Department of Epidemiology, Public Health faculty, Jimma University, Jimma, Ethiopia; 7 Department of Global Health and Population, Harvard T.H. Chan School of Public Health, Boston, MA 02115 USA

**Keywords:** Hepatitis B virus, Vaccines, Mother-to-child transmission, Birth dose of HBV vaccine, Cost-effectiveness analysis, Sub-Saharan Africa, Ethiopia

## Abstract

**Background:**

Hepatitis B virus (HBV) infection is an important cause of morbidity and mortality with a very high burden in Africa. The risk of developing chronic infection is marked if the infection is acquired perinatally, which is largely preventable through a birth dose of HBV vaccine. We examined the cost-effectiveness of a birth dose of HBV vaccine in a medical setting in Ethiopia.

**Methods:**

We constructed a decision analytic model with a Markov process to estimate the costs and effects of a birth dose of HBV vaccine (the intervention), compared with current practices in Ethiopia. Current practice is pentavalent vaccination (DPT-HiB-HepB) administered at 6, 10 and 14 weeks after birth. We used disability-adjusted life years (DALYs) averted to quantify the health benefits while the costs of the intervention were expressed in 2018 USD. Analyses were based on Ethiopian epidemiological, demographic and cost data when available; otherwise we used a thorough literature review, in particular for assigning transition probabilities.

**Results:**

In Ethiopia, where the prevalence of HBV among pregnant women is 5%, adding a birth dose of HBV vaccine would present an incremental cost-effectiveness ratio (ICER) of USD 110 per DALY averted. The estimated ICER compares very favorably with a willingness-to-pay level of 0.31 times gross domestic product per capita (about USD 240 in 2018) in Ethiopia. Our ICER estimates were robust over a wide range of epidemiologic, vaccine effectiveness, vaccine coverage and cost parameter inputs.

**Conclusions:**

Based on our cost-effectiveness findings, introducing a birth dose of HBV vaccine in Ethiopia would likely be highly cost-effective. Such evidence could help guide policymakers in considering including HBV vaccine into Ethiopia’s essential health services package.

## Background

Hepatitis B virus (HBV) infection remains an important cause of morbidity and mortality globally. According to 2015 estimates by the World Health Organization (WHO), about 260 million individuals (3.5% of the world’s population) were living with chronic HBV infection in the world [1]. Most HBV infections (68%) occurred in the African and Western Pacific regions where the HBV prevalence was the highest (6.1% in the African region and 6.2% in Western Pacific among the general population, respectively) [1]. In 2015, viral hepatitis led to 1.34 million deaths globally, of which 66% were resulting from complications of chronic HBV infection such as cirrhosis (53%) and hepatocellular carcinoma (34%) [1].

The prevalence of HBV infection is estimated at 7.4% among the general population of Ethiopia, which is at the highest end of intermediate endemicity^1^ [2]. Likewise, according to a meta-analysis of prevalence studies in Ethiopia, the prevalence of HBV infection among pregnant women was estimated at 4.7% that is lower than estimates from other African countries such as Nigeria, Cameroon and Ghana where the prevalence of HBV infection among pregnant women ranged from 9.8 to 14.1% [3]. Pregnant women infected with HBV can potentially transmit the virus to their infants usually during birth. The risk of transmission to the newborn is largely dependent on the presence or absence of the hepatitis B virus envelope antigen (HBeAg) in HBV-infected pregnant women. In Africa, infants born to mothers who are positive for both hepatitis B surface antigen (HBsAg) and HBeAg carry a 28% risk of transmission with a lower risk (8%) in mothers who have lost HBeAg [4]. A study on the risk of mother to child transmission (MTCT) of HBV in Ethiopia has shown a higher transmission rate [5]. Perinatally infected infants are at the highest risk of chronic HBV infection that occurs in 80–90% of infected infants [6].

Measures to control viral hepatitis have progressively increased globally [1]. Most countries including Ethiopia have introduced hepatitis B vaccine into their routine infant immunization programs. The birth dose of hepatitis B (HepB-BD) vaccine is the main modality in the prevention of mother to child HBV transmission. Ideally, the birth dose should be given within 24 h of birth but can still be partially effective even if given beyond, its effectiveness diminishing with the passage of time [7]. Unfortunately, as of 2015, only 10% of African countries had introduced HepB-BD vaccine, and it is not yet introduced in Ethiopia [1]. Furthermore, other preventive measures such as hepatitis B immunoglobulin or medications for the treatment of HBV infection are not yet part of Ethiopia’s essential health service package [8]. In the absence of universal HepB-BD vaccine or other effective interventions, perinatally acquired HB infection remains a major cause of chronic liver disease when infected children reach adulthood [9].

The fight against viral hepatitis is gathering momentum globally. Combating hepatitis is included in the Sustainable Development Goals (SDG 3.3) [10]. The World Health Assembly in 2016 adopted a Global Health Sector Strategy on viral hepatitis with elimination as its overarching goal [1]. In line with the global response, Ethiopia’s Federal Ministry of Health (FMoH) has developed guidelines aiming at scaling up viral hepatitis preventive measures, and standardized screening and management of patients with viral hepatitis toward improved outcome [11].

Evidence on the cost-effectiveness of HepB-BD vaccine is scarce in sub-Saharan Africa. A cost-effectiveness analysis (CEA) of adding HepB-BD vaccine in Mozambique has shown that it could be highly cost-effective within the Mozambique health system [12]. Yet, CEA results may vary with the setting, the epidemiology of HBV and intervention costs. Therefore, in this paper, we evaluate the incremental cost-effectiveness of adding HepB-BD vaccine to the three-dose regimen given to infants at 6, 10 and 14 weeks after birth. The evidence we generate serve as a useful input for Ethiopian policymakers in considering including HepB-BD vaccine into the national essential health services package.

## Methods

**Fig. 1:**
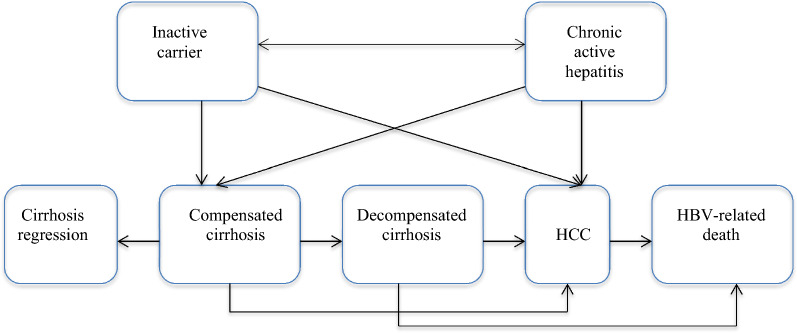
Markov process showing the different health states

We constructed a decision analytic model with a Markov process (Additional file 1 and Fig. 1) to estimate intervention costs and health impact of an infected individual over a lifetime. We used TreeAge Pro 2018 software for the analysis. As demonstrated in the Additional file 1 and Fig. 1, the model reflects the natural history of perinatally acquired HBV-infection [13]. We compared two strategies. In the novel strategy (HepB-BD vaccine-plus) all infants receive HepB-BD vaccine (monovalent) within 24 h of delivery and continue on with the pentavalent vaccine (DPT-HiB-HepB) series starting at the age of 6 weeks. We assumed 50% of the target birth cohort will be born in health care facilities and receive the birth dose in a medical setting; following the latest skilled birth attendance rate in Ethiopia [14]. We further assumed that the government rolls-out HepB-BD vaccine to the whole birth cohort and therefore incur costs but vaccine effectiveness would vary depending on the skilled birth attendance rate in Ethiopia. In the current strategy, the birth cohort will only receive the existing pentavalent vaccination schedule at 6, 10 and 14 weeks after birth.

In the model, we assumed HepB-BD vaccine would prevent against MTCT of HBV infection during birth/delivery (vertical transmission), otherwise the two strategies would have similar efficacy on infections that occur later in life (horizontal transmissions). We used data on HBV prevalence among pregnant women, vaccine effectiveness, and the risk of perinatal transmission to calculate the percentage of children born with HBV infection. Infected infants either develop acute symptomatic infection with a risk of fulminant hepatitis or remain asymptomatic (Additional file 1). Even though symptomatic acute infections occur less frequently in infants, we accounted for their costs and health consequences in our model (see Additional file 1) [15, 16]. Perinatally infected infants develop acute symptomatic infections early in life, therefore we calculated disability-adjusted life years (DALYs)^2^ (based on life expectancy at birth adjusted for health state valuations from WHO-CHOICE) for only fatal cases and did not account for the lost quality of life for the duration of acute illness [17, 18]. The majority (90%) of the asymptomatic cases and about 33% of those surviving fulminant hepatitis will develop chronic HBV infection [4, 15]. Most perinatally infected individuals enter the immunoactive phase and develop HBeAg positive chronic hepatitis with elevated liver enzyme (alanine aminotransferase) levels only after 10–30 years of infection [19]. Therefore, we started the Markov process at age 20 years. Individuals with chronic HBV infection are simulated within the Markov process either as an inactive carrier or with chronic active hepatitis (CAH). Individuals who do not require antiviral therapy are considered inactive carriers and CAH are those who fulfill the treatment criteria (irrespective of the HBeAg status). A study in Ethiopia has evaluated treatment eligibility and response to antiviral management of chronic hepatitis B infection [20]. In this study, among individuals 18–25 years of age with chronic HBV infection, nearly a quarter of patients (25%) were eligible to antiviral treatment (ALT > 80 U/L and viral load > 2000 IU/mL). Therefore, in our model 75% of the individuals with chronic HBV infection began the Markov process as inactive carriers while 25% as CAH. Even though there are studies that document clearance of HBsAg (in 6–17% of the cases) in Caucasian children who acquired chronic HBV infection horizontally, such data for perinatally infected children in sub-Saharan Africa were lacking where there is limited access to antiviral treatment [21, 22]. Therefore, our model did not account for state transitions from either inactive carriers or CAH to HBsAg clearance (no infection).

We ran the Markov process for 51 cycles, which corresponds to the average life expectancy of both males and females at age 20 years in Ethiopia [17]. Every Markov cycle lasts 1 year allowing infected individuals to pass through different morbid states based on their transition probabilities. In each annual cycle, infected individuals could incur costs related to medical care and health outcomes depending on their health state. Subsequently for each strategy, the costs and effects accrued in each of the decision trees and the Markov cycles are added and compared to calculate the incremental cost-effectiveness ratio (ICER).

### Epidemiologic, probability and effectiveness data

The overall prevalence of HBsAg among pregnant women in Ethiopia was estimated at 4.7% [3]. The risk of perinatal transmission varies by the maternal HBeAg status, where HBeAg-positive mothers carry a higher risk of transmission [4]. Prevalence of HBeAg among HBsAg-positive pregnant women in Ethiopia is unknown; therefore we used the mean prevalence from several sub-Saharan African countries [4]. Table 1 summarizes epidemiologic and probability data inputs used in our model. Individuals infected with HBV may develop a spectrum of disease conditions with different probabilities (see Fig. 1 and Additional file 1). Local data on transition probabilities among different health states were not available. Therefore we used data from settings that are similar to the Ethiopian context when available (Table 1) [23, 24]. Similar to what has been used in acute symptomatic infections, DALYs averted were the measure of effectiveness for chronic HBV states, inline with WHO recommendations since chronic HBV infection results in both premature mortality and morbidity [23, 25]. Evidence on disability weights for the different disease states were gathered from the Global Burden of Diseases (GBD) study database but other sources were also sought when such data were not available in the GBD database (Table 2) [24, 26].

**Table 1:** Epidemiologic parameters and annual transition probabilities used in the model

Parameter	Base assumptions	Range for sensitivity analysis	Source
Prevalence of HBsAg among pregnant women	0.047	0.025–0.1	[3]
Prevalence of HBeAg among HBsAg positive mothers	0.116	0.05–0.25	[4]
Risk of perinatal transmission (HBeAg +)	0.279	0.1–0.6	[4]
Risk of perinatal transmission (HBeAg−)	0.08	0.02–0.29	[4]
Acute symptomatic cases (for children of HBeAg + mothers)	0.01	–	[4]
Acute symptomatic cases (for children of HBeAg − mothers)	0.05	–	[23]
Fulminant infections among symptomatic cases	0.001	–	[15]
Risk of infant death from fulminant infection	0.7	–	[15]
Risk of infant chronic infection among survivors of fulminant infection	0.33	–	[15]
Risk of chronic HBV infection after perinatal infection	0.9	–	[4]
Chronic HBV infected individuals requiring antiviral treatment (CAH)	0.25	–	[20]
Inactive carriers among individuals with chronic HBV infection	0.75	–	[20]
Annual transition probabilities and disease related mortality			
Inactive carrier to			
CAH	0.03	–	[23]
Compensated cirrhosis	0.007	–	[24]
HCC	0.0006	–	[24]
CAH to^a^			[24]
Inactive carrier	0.55	0.3–0.7	[24]
Compensated cirrhosis	0.0024	0.0012–0.0036	[24]
HCC	0.003	0.002–0.007	[24]
Compensated cirrhosis to			
Decompensated cirrhosis	0.035	–	[23]
HCC	0.033	–	[23]
Regression	0.24	–	[24]
Decompensated cirrhosis to			
Disease specific death	0.28	–	[23]
HCC	0.15	–	[23]
HCC to			
Disease specific death	0.29	–	[23]
Vaccine effectiveness	0.72	0.6–0.8	[27]
Vaccine utilization	0.5	0.1–0.66^b^	[14]

^a^In our model, patients with chronic active hepatitis (CAH) are eligible for antiviral treatment therefore we used transition probabilities of treated patients

^b^The highest vaccine utilization rate was based on administrative report for facility delivery in Ethiopia

**Table 2:** Disability weights for different disease states used in the model

Disease state	Disability weight	Source
Inactive carrier	0.0	[26]
Chronic active hepatitis^a^	0.01	[24]
Compensated cirrhosis	0.0	[26]
Decompensated cirrhosis	0.178	[26]
Hepatocellular carcinoma (HCC)^b^		
Diagnosis and primary treatment	0.288	[26]
Metastasis	0.451	[26]
Terminal	0.54	[26]

^a^Fan L, presented quality adjusted life years and we simply subtracted these values from one to compute disability weights

^b^We just took the average disability weights of the different HCC stages to calculate the annual HCC disability weight of 0.43

Vaccine effectiveness data based on a randomized controlled trial (RCT) were not available locally and from other comparable sub-Saharan African countries. Therefore we used data from other settings that were based on a systematic review of the efficacy of hepatitis B immunization for newborn infants of HBsAg-positive mothers where the protective efficacy of the vaccine was 72% (95% CI 60 to 80%) in preventing perinatal HBV transmission [27]. Vaccine adverse effects that are usually mild were not factored into the model [6]. Deaths due to other causes (background mortality) were integrated in the model using the World Health Organization’s life tables for Ethiopia [17].

### Costs

Costs were estimated from a health provider perspective and only included direct medical costs. We estimated the incremental cost of introducing HepB-BD vaccine in its monovalent form. We preferred the monovalent form of the vaccine in order to minimize wastage rates. Both recurrent and capital costs were included using an ingredients-based approach following WHO guidelines [28]. Recurrent costs included costs of vaccines, syringes, safety boxes, transport and maintenance, and cold chain storage while social mobilization and training costs were under capital costs (Table 3). We used the latest UNICEF price data for vaccines, auto-disable (AD) syringes and safety box adjusted for wastage rates and freight costs [29, 30]. Data on wastage rates and freight costs were retrieved from a study on economic evaluation of HBV vaccine in low-income countries [23]. Local data on other aspects of recurrent costs (transport and maintenance and cold chain storage) and capital costs for HepB-BD vaccine were not available therefore we used 2008 estimates from Mozambique adjusted to the 2018 USD values [12, 31].

**Table 3:** Vaccination cost estimates and intervention costs in 2018 US$

Parameter	Base assumptions	Range for sensitivity analysis	Source
Vaccination cost			
Recurrent costs			
Vaccine^a^	0.25200	–	[23, 29]
Syringes and safety box^b^	0.04824	–	[23, 30]
Transport and maintenance	0.02206	–	[12, 31]
Cold chain storage	0.02213	–	[12, 31]
Capital costs			
Social mobilization	0.05276	–	[12, 31]
Training	0.34532	–	[12, 31]
Total average costs per vaccinated child	0.74251	0.50–1.00	
Medical care costs (US$)			
Initial assessment and diagnosis cost	113.16	56.58–226.32	Local data
Drug treatment	60.35	30.17–120.70	[32, 33]
Monitoring on treatment	37.00	18.50–74.00	Local data
Monitoring without treatment	18.50	9.25–37.00	Local data
Average annual cost of hospital admission for decompensated cirrhosis	153.88	76.94–307.76	[34]
Average annual cost of hospital admission for hepatocellular carcinoma	153.88	76.94–307.76	[34]
Treatment cost of symptomatic acute infection	40.00	20.00–80.00	Assumptions
Treatment cost of fulminant hepatitis	200.00	100.00–400.00	Assumptions

^a^Vaccine wastage rate was assumed to be 20% and a freight rate of 6%

^b^Wastage and freight rates were 10% and 15%, respectively

The medical care costs included initial assessment and diagnosis costs, antiviral drug costs, costs associated with monitoring those on treatment and not on treatment, and cost of managing decompensated cirrhosis (DCC) and hepatocellular carcinoma (HCC) (Table 3). In order to calculate the costs of initial assessment and diagnosis, we used the national guidelines on viral hepatitis to identify required laboratory tests and imaging modalities and frequency of health care visits [11]. We collected laboratory cost data from local sources. The median supplier price for the antiviral drug of choice (Tenofovir) was used to compute for annual drug cost after accounting for transportation costs [32, 33]. Hospital admission cost data for DCC and HCC were not locally available; therefore we used estimates from The Gambia adjusted to the 2018 USD values [31, 34]. Costs for acute symptomatic conditions were based on expert estimates. All costs were expressed in 2018 USD. Both future costs and health outcomes were discounted at 3% annual rate following WHO recommendations [25].

### Sensitivity analyses

We conducted a series of one-way sensitivity analyses where we varied key input parameters one at a time over plausible ranges to test the robustness of our findings (Table 1). Based on the findings in a one-way sensitivity analysis, we proceeded and conducted two-way and three-way sensitivity analyses for parameters that are likely to change the result in a critical way. Furthermore, a multivariate sensitivity analysis was conducted using Monte Carlo simulations with n = 10,000 simulation runs. We varied all key parameters (vaccine effectiveness, vaccine utilization, risk of perinatal transmission in HBeAg-mothers, risk of perinatal transmission in HBeAg + mothers, prevalence of HBV infection among mothers, cost of medical care, average cost per vaccinated child, prevalence of HBeAg in pregnant women, transition probability of CAH to inactive carrier state) simultaneously [35]. Lastly, we also ran the model without discounting future health benefits or costs (one at a time), and without discounting both health benefits and costs [35].

## Results

Compared to the current strategy, an additional HepB-BD vaccine would have an incremental cost-effectiveness ratio of 110 USD per DALY averted (Table 4). Leaving effects and both effects and costs undiscounted decreased the ICER to 67 USD and 49 USD per DALY averted, respectively.

**Table 4:** Cost, effectiveness and incremental cost effectiveness ratio (ICER) of an additional birth dose of HB vaccine

Strategy	Cost (US$)	Incremental costs	Effects (DALYs averted)	Incremental effects (DALYs averted)	ICER
Without birth dose	4.0243		0.001417		
With birth dose	4.3538	0.3295	0.004117	0.00300	110

The one-way sensitivity analyses showed that the ICERs would range from cost savings to maxima of USD625 per DALY averted. The highest ICER was observed for a 10% vaccine utilization, followed by a 2% risk of perinatal transmission when the mother was HBeAg negative and a 2.5% prevalence of HBV infection among mothers with an ICER of USD373 to 328 per DALY averted (Table 5). With two-way sensitivity analyses, we found that various combinations of vaccine effectiveness with other parameters would change the ICER in a substantial way as follows (Table 5). ‘’Vaccine effectiveness’ and ‘average cost per vaccinated child’ an ICER of 262; ‘vaccine effectiveness’ and ‘prevalence of HBV infection among mothers’ an ICER of 421; ‘vaccine effectiveness’ and ‘risk of perinatal transmission when mother was HBeAg negative’ an ICER of USD475 per DALY averted; and ‘Vaccine effectiveness’ and ‘vaccine utilization’ would have ICER of USD663 per DALY averted. The three-way sensitivity analyses also showed that most parameter variations resulted in an ICER value of < USD485 per DALY averted except for some combinations of the following critical parameter inputs: vaccine utilization, risk of perinatal transmission when mother was HBeAg positive, risk of perinatal transmission when mother was HBeAg negative, average cost per vaccinated child and prevalence of HBV infection among mothers with an ICER ranging from USD947 to 1493 per DALY averted. On the contrary, a combination of the following parameters: risk of perinatal transmission when mother was HBeAg negative, annual cost of CAH and prevalence of HBV infection among mothers at their favorable parameter inputs became cost savings (hence ICER < 0). The univariate sensitivity analysis demonstrated that introducing HepB-BD vaccine in Ethiopia is less likely to be highly cost-effective (at a threshold of 0.31 times the GDP per capita in Ethiopia, USD240 in 2018) when the vaccine utilization drops below 35% [36, 37].

**Table 5:** Results of one-way and two-way sensitivity analyses

One-way			Two-way	
Parameters	Range for sensitivity analysis	ICER (US$ per DALY averted)	Parameters with range for sensitivity analysis	ICER (US$ per DALY averted)
Vaccine effectiveness	80%	93	Vaccine effectiveness (60–80%) + Vaccine utilization (10–66%)	88
	60%	169		663
Vaccine utilization	66%	90	Vaccine effectiveness (60–80%) + Risk of perinatal transmission (HBeAg −) (2%–29%)	CS
	10%	625		475
Risk of perinatal transmission (HBeAg −)	29%	CS	Vaccine effectiveness (60–80%) + Prevalence of HBV infection among mothers (2.5–10%) (10–66%)	CS
	2%	373		421
Prevalence of HBV infection among mothers	10%	CS	Vaccine effectiveness (60%–80%) + Cost of medical care (from no cost to twice the cost of morbid states) (10–66%)	CS
	2.5%	328		207
Cost of medical care	Twice the cost	CS	Vaccine effectiveness (60–80%) + Average cost per vaccinated child ($0.5–1.0) (10–66%)	13
	No cost	158		262
Average cost per vaccinated child	$0.5	29	Vaccine effectiveness (60–80%) + Risk of perinatal transmission (HBeAg +) (14–56%)	32
	$1.0	195		214
Risk of prinatal transmission (HBeAg +)	56%	51	Vaccine effectiveness (60–80%) + Prevalence of HBeAg in pregnant women (5–25%) (10–66%)	40
	14%	156		202
Prevalence of HBeAg in pregnant women	25%	59	Vaccine effectiveness (60–80%) + Transition prob. of CAH to inactive carrier state (30–70%) (10–66%)	35
	5%	146		174
Transition prob. of CAH to inactive carrier state	30%	60		
	70%	124		

*CS* cost saving

**Fig. 2:**
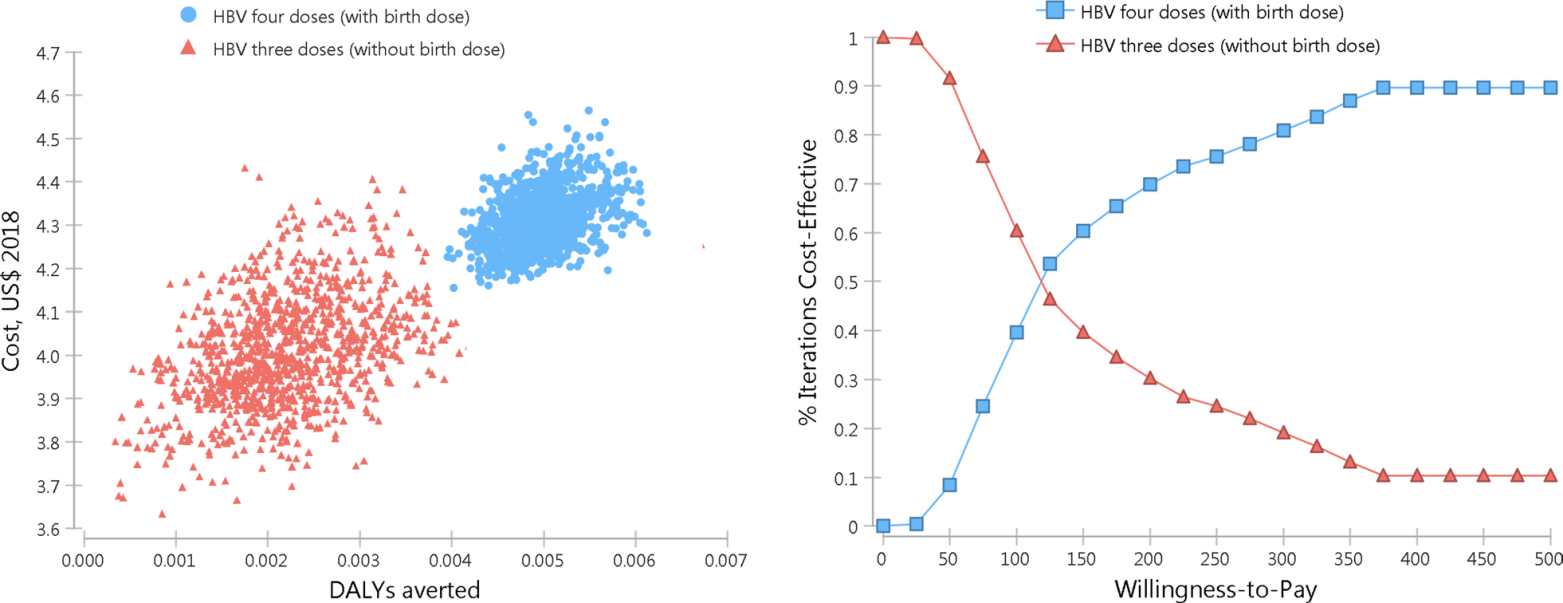
Probabilistic sensitivity analysis (n = 10,000 simulations)

Figure 2 shows the results of the probabilistic sensitivity analysis and the uncertainty surrounding our cost-effectiveness estimates, with overlapping costs and effectiveness ranges. Regardless, the distribution pattern is relatively distinct with higher costs and effectiveness for the novel strategy (with birth dose). The willingness-to-pay (WTP) threshold has an impact on the probability of intervention being cost-effective. At WTP threshold of greater than US$110 per DALYs averted, the four-dose regimen (with birth dose) is most likely to be cost-effective.

For a birth cohort of 3.34 million in 2018 in Ethiopia, we calculated a total of 10,020 DALYs would be averted at a cost of nearly USD 2.5 million for HBV vaccine introduction (without accounting for the cost saving from prevention of disease states) [38].

## Discussion

We conducted a cost-effectiveness analysis of HepB-BD vaccine in its monovalent form (compared with current practices) in Ethiopia and found incremental cost-effectiveness ratio of USD110 per DALY averted. The ICER for HepB-BD is lower than the willingness-to-pay thresholds suggested by Ochalek J et al. < 0.31 times the GDP per capita in Ethiopia [37]. This makes investing in HepB-BD vaccine highly cost-effective in an Ethiopian setting, especially in light of the cost-effectiveness of comparable interventions for low-income countries in sub-Saharan Africa [39–41]. Our cost-effectiveness findings remained robust to a number of sensitivity analyses (e.g. Table 5). Among the key parameters tested in sensitivity analyses, vaccine utilization was the main driver behind the cost-effectiveness results, followed by risk of perinatal transmission associated with HBeAg status, average cost per vaccinated child, prevalence of HBV infection among mothers, and treatment cost of CAH. In addition, our findings are comparable to those of other studies that studied the cost-effectiveness of birth dose of HB vaccine in Mozambique [12, 42].

Despite demonstrating that HepB-BD vaccine is highly cost-effective in an Ethiopian setting, our analysis presents a number of important limitations. First, local data on vaccine effectiveness, health state transition probabilities, and the cost of different health states were not readily available which may decrease the accuracy behind our ICER estimates. Second, we only assessed the cost of tenofovir as the treatment of choice in our analysis but using other drugs such as entecavir might be an alternative cost-effective therapy [43]. Treatment of chronic HBV infection with entecavir consumes more resources as compared to treatment with tenofovir, which might have resulted in a less cost-effective estimate in our analysis [44]. Due to lack of community-based data on treatment eligibility, we used a study conducted in a hospital setting, which might over represent symptomatic cases [20]. Third, some aspects of vaccine delivery costs (such as transport and maintenance, cold chain storage and capital costs) were based on estimates from other settings that may not reflect the reality in Ethiopia. Fourth, another important limitation is associated with limiting vaccine delivery in the medical setting. Skilled birth attendance rate is low and many women in Ethiopia give birth at home without skilled assistance, especially those women residing in rural areas [14]. Making HBV vaccine accessible to the whole population might require a community-level engagement, which might have additional cost implications. Cost estimates of such outreach strategies were not available and not included in our model. Including the cost of outreach strategies in the analysis may result in higher ICER related to additional resource requirements such as transport, fuel and staff allowance [45]. We also assumed that additional health professionals would not be required to administer the birth dose of HB vaccine and therefore we have not included related costs. Even though health work force per population is low in Ethiopia, recent evidence suggests that there is underutilization of available health workforce in the country [46, 47]. Lastly, there may be additional important benefits such as the equity benefits of vaccinations including the prevention of expensive out-of-pocket medical treatment for liver cancer and associated medical impoverishment into the future [48].

Successful implementation of HepB-BD vaccine is largely dependent on the timing of its administration. Timely administration (within 24 h of birth) of the vaccine may require integration with maternal and child health (MCH) programs rather than through the Expanded Program on Immunization (EPI). The suggested change in the delivery of HepB-BD vaccine requires redesigning the MCH (immediate post-partum services) and EPI programs and could include other birth dose vaccines such as Bacille Calmette-Guérin (BCG) and the birth dose of oral polio vaccine (OPV-0). This will entail not only additional work on midwives (or other health personnel rendering immediate post-partum services) but also logistic implications for vaccine storage and delivery within the maternity setting. Further, this might require training of health personnel and awareness-raising campaigns nationally in Ethiopia. With additional efforts on social mobilization, the introduction of HepB-BD vaccine may positively contribute to the improvement in the coverage of skilled birth attendance and other birth dose vaccines in Ethiopia.

Besides, resources for health care are scarce in Ethiopia. The total health expenditure per capita in Ethiopia was about US$33 for the years 2016/2017 [49]. The annual budget required to introduce the birth dose of HBV vaccine in Ethiopia costs an additional 0.023 US$ per capita which is nearly 0.1% of the 2016/17 annual total health expenditure (3.1 billion US$) for Ethiopia. In the last decade, Ethiopia has enjoyed a substantial economic development and aspires to be a middle-income country by 2035 [50]. Along with the economic development, the government of Ethiopia envisions to attain population health status commensurate with the best performing middle-income countries [50]. Realization of such ambitious targets requires the government’s commitment for more health care resource allocations.

## Conclusions

Birth dose of HB vaccine is one of the most important preventive strategies recommended by the WHO and the national viral hepatitis control and prevention guidelines in Ethiopia. Given the formidable resource constraints in low-income countries such as Ethiopia, evidence on cost-effectiveness of interventions is vital towards setting priorities. Currently, the Ethiopian government is revising its essential health services package (EHSP). Given the substantial health dividend in investing in the birth dose of HB vaccine in Ethiopia and the fact that it is highly cost-effective, it should be considered for inclusion in the Ethiopia’s EHSP.

## Supplementary information

**Additional file 1.** Decision tree structure.

## Data Availability

The dataset supporting the conclusions in this article have been provided in Tables 1, 2, 3.

## Abbreviations

ALT: Alanine aminotransferase
CAH: Chronic active hepatitis
CEA: Cost-effectiveness analysis
DALYs: Disability adjusted life years
DCC: Decompensated cirrhosis
EPI: Expanded program on immunization
FMOH: Federal ministry of health
HBeAg: Hepatitis B virus envelope antigen
HBsAg: Hepatitis B surface antigen
HBV: Hepatitis B virus
HCC: Hepatocellular carcinoma
HepB-BD: Birth dose of hepatitis B
ICER: Incremental cost-effectiveness ratio
MCH: Maternal and child health
MTCT: Mother to child transmission
UNICEF: United nation children’s fund
WHO: World Health Organization

## Footnotes

1 The endemicity of hepatitis B virus is determined by the prevalence of HBsAg in the general population of a defined geographic area: prevalence of HBsAg > 8% is high endemicity, 2–7% intermediate endemicity, and HBsAg prevalence < 2% is low endemicity [6].

2 Most of the deaths from perinatally acquired acute fulminant hepatitis occur during infancy. Using a life table for Ethiopia, adjusted for health state valuations, we computed the DALYs lost associated with each death (54.3 years). This value was used in the model to quantify the DALYs lost associated with deaths due to fulminant hepatitis.
