# Supplementary material for: Introduction of birth dose of hepatitis B virus vaccine to the immunization program in Ethiopia: an economic evaluation
Source: Cost Eff Resour Alloc. 2020 Jul 22;18:23. doi: 10.1186/s12962-020-00219-7 (PMC7374878; doi:10.1186/s12962-020-00219-7)

Additional file 1: Decision tree structure.

PW-HBsAg+

Strategy X

PW-HBsAg-

PW-HBeAg+

PW-HBeAg-

Infant-HBsAg-

Infant-HBsAg+

No symptom

Symptom

Resolve

Chronic


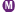


Fulminant

Non-fulminant

Resolve

Chronic


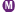


Resolve

Die

Chronic


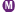


Infant-HBsAg-

Infant-HBsAg+

No symptom

Symptom

Resolve

Chronic


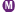


Fulminant

Non-fulminant

Resolve

Chronic


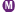


Resolve

Die

Chronic


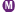

Supplement: Supplementary file 1 — Additional file 1. Decision tree structure. [file 12962_2020_219_MOESM1_ESM.docx]
